# Supplementary material for: Electrosprayed Alginate Nanoparticles as CRISPR Plasmid DNA Delivery Carrier: Preparation, Optimization, and Characterization
Source: Pharmaceuticals (Basel). 2020 Jul 22;13(8):158. doi: 10.3390/ph13080158 (PMC7465179; doi:10.3390/ph13080158)
Supplement: Supplementary file 1 [file pharmaceuticals-13-00158-s001.pdf]

SUPPLEMENTARY MATERIALS

1. Selection of CRISPR pDNA: ALG ratio

More than 99% encapsulation efficiency (EE) was obtained by using 1:100 and 10:100 pDNA:ALG (w:w) ratio. Since 1:100 and 10:100 pDNA:ALG solutions were not significantly different, further cytotoxicity study was conducted to determine optimum ratio. Interestingly, CRISPR NPs with 10:100 ratio showed no cytotoxicity as the cell metabolic activity was 132.39±5.25% at NPs concentrations (125 µg/mL) after 48 h, compared to 85.53%±1.44% for the 1:100 ratio at the same NPs concentration. Therefore, 10:100 pDNA/ALG ratio was selected to prepare the nanoparticles.

2. Optimization of Process Variables

The regression equation was

Particle Size (nm)= 509.00 + 24.33 Flow Rate (ml/hr)_0.1 + 21.89 Flow Rate (ml/hr)_0.3
- 46.22 Flow Rate (ml/hr)_0.5 + 169.78 Applied Voltage (Kv)_9.5
+ 78.89 Applied Voltage (Kv)_11.0 - 248.67 Applied Voltage (Kv)_12.5
+ 57.6 Flow Rate (ml/hr)*Applied Voltage (Kv)_0.1 9.5
- 9.2 Flow Rate (ml/hr)*Applied Voltage (Kv)_0.1 11.0
- 48.3 Flow Rate (ml/hr)*Applied Voltage (Kv)_0.1 12.5
- 9.3 Flow Rate (ml/hr)*Applied Voltage (Kv)_0.3 9.5
- 11.1 Flow Rate (ml/hr)*Applied Voltage (Kv)_0.3 11.0
+ 20.4 Flow Rate (ml/hr)*Applied Voltage (Kv)_0.3 12.5
- 48.2 Flow Rate (ml/hr)*Applied Voltage (Kv)_0.5 9.5
+ 20.3 Flow Rate (ml/hr)*Applied Voltage (Kv)_0.5 11.0
+ 27.9 Flow Rate (ml/hr)*Applied Voltage (Kv)_0.5 12.5 ……………………………………Eq (3)

3. Encapsulation Efficiency

In the current study, the alginate grade used was high stiffness gelation 1L-6G with high-G content. The EE was 99.94±0.10%. In primary experiments using different alginate grades, the EE varied from 84.40% to 93.30% for Manucol LF (low molecular weight, high M, 10-40 mPa.S) and Manugel DMB (high molecular weight, high G, 300 mPa S), respectively.

4. Cumarin-6 Labelled CRISPR ALG NPs Properties

Cumarin-6 labelLed CRISPR ALG NPs showed a spherical morphology with smooth surface. Their particle size, PDI and zeta potential was 218.33 ± 28.02 nm, 0.48 ± 0.171, and -4.22 ± 0.28 mV, respectively. No significant difference in properties was observed between CRISPR ALG NPs and cumarin-6 labeled counterpart ($p>0.05$).

Figure S1. TEM image of cumarin-6 labeled CRISPR ALG NPs

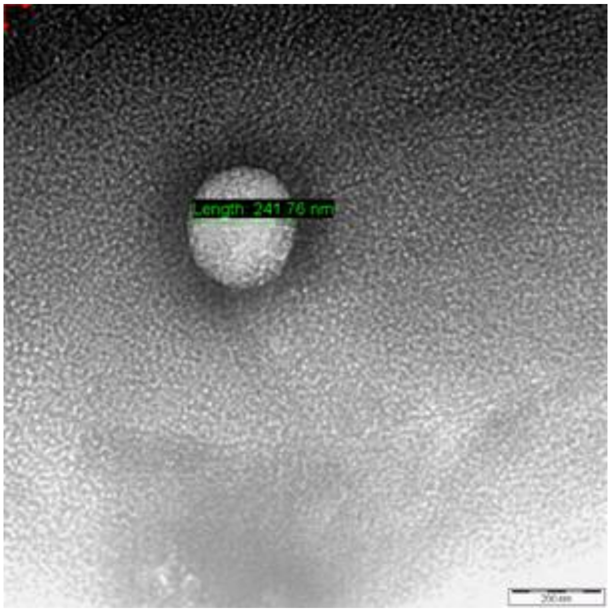
Length: 241.76 nm
200 nm
